# Temporal evolution of work accidents in Santa Catarina construction industry

**DOI:** 10.47626/1679-4435-2024-1297

**Published:** 2025-07-13

**Authors:** Marcos Alano Gomes de Carvalho, Tales Pereira Costa

**Affiliations:** 1 Departamento de Medicina, Universidade do Sul de Santa Catarina, Tubarão, SC, Brasil

**Keywords:** construction industry, accident, occupational, time series, construção civil, acidente de trabalho, séries temporais

## Abstract

Civil construction, a department that employs many workers, occupies a third of the industrial department and contributes in 6.2% of the Brazilian gross domestic product GDP, is among the economic activities with the highest risk of work-related accidents. In this perspective, the Regulatory Norms of the Consolidation of Labor Laws define standards for the preservation of workers’ health. This article aims to analyze the temporal evolution of work-related accidents in the construction industry in the state of Santa Catarina (2011-2020). This is an ecological observational study, with time series analysis. The studied population consisted of building construction workers (Classificação Nacional de Atividades Econômicas 41) linked to the General Social Security System, who suffered work accidents, in the state of Santa Catarina, in the years 2011 to 2020. The results obtained indicated the influence of the recurring economic crises that occurred in the period, which had a direct impact on the hiring of labor and the level of economic activity, and consequently, on accident rates. Regarding the consequences of work-related accidents in Classificação Nacional de Atividades Econômicas 41, there was a trend of growth in the rates of leaves due to disability of less than 15 days and a reduction in the rates of incapacity greater than 15 days. This result may be related to the creation, by Social Security, of economic mechanisms that induce more effective work safety actions by companies. There was a considerable reduction in the risk of accidents at work in the construction of buildings in the state of Santa Catarina in the analyzed period.

## Abstract (pt)

O setor da construção civil, que emprega muitos trabalhadores e representa um terço do setor industrial, contribui com 6,2% do Produto Interno Bruto brasileiro e está entre as atividades econômicas com maior risco de acidentes de trabalho. Nessa perspectiva, as Normas Regulamentadoras da Consolidação das Leis do Trabalho definem padrões para preservação da saúde dos trabalhadores. Este artigo objetiva analisar a evolução temporal dos acidentes de trabalho na indústria da construção civil no estado de Santa Catarina, no período de 2011-2020. Trata-se de um estudo observacional, de tipo ecológico, com análise de séries temporais. A população estudada foi composta por trabalhadores da construção de edifícios – Classificação Nacional de Atividades Econômicas CNAE 41 – vinculados ao Regime Geral de Previdência Social, que sofreram acidentes de trabalho, no estado de Santa Catarina, nos anos de 2011-2020. Os resultados obtidos indicaram a influência das recorrentes crises econômicas ocorridas no período, que impactaram diretamente a contratação de mão de obra e o nível da atividade econômica e, consequentemente, as taxas de acidentes. Em relação às consequências de acidentes de trabalho na Classificação Nacional de Atividades Econômicas 41, houve tendência de crescimento nas taxas de afastamentos por incapacidade inferior a 15 dias e de redução nas taxas de incapacidade superior a 15 dias. Este resultado pode estar relacionado à criação, pela Previdência Social, de mecanismos econômicos indutores de ações mais efetivas de segurança do trabalho por parte das empresas. Houve redução considerável no risco de ocorrência de acidentes de trabalho na construção de edifícios no estado de Santa Catarina no período analisado.

## INTRODUCTION

According to literature, occupational medicine, as a medical specialty, began in England in the first half of the 19th century. At the start of the Industrial Revolution, workers were subjected to intense, inhumane production processes, leading to the overexploitation of labor. This period was marked by poor health and safety conditions in the workplace. The frequent occurrence of serious accidents and fatal work-related illnesses led to the creation of regulations and laws aimed at protecting workers’ health.^1^

In Brazil, workers’ health and safety are regulated by Ordinance no. 3,214, dated June 8, 1978. This ordinance established the Regulatory Standards (Normas Regulamentadoras, NRs) of the Consolidation of Labor Laws related to Occupational Safety and Health.^2^ NR-07 requires all employers and institutions that hire employees to create and implement the Occupational Health Medical Control Program (Programa de Controle Médico de Saúde Ocupacional, PCMSO). The goal of this program is to promote and protect the overall health of all workers.^3^

According to data from the Brazilian Continuous National Household Sample Survey (Pesquisa Nacional por Amostragem de Domicílio Contínua, PNAD Contínua), a household survey conducted by the Brazilian Institute of Geography and Statistics (Instituto Brasileiro de Geografia e Estatística, IBGE) that includes both formal and informal workers, the highest number of workers in the construction industry was recorded in 2014, with 8.1 million people employed in the sector. The lowest number occurred in 2020, due to the COVID-19 pandemic, with 5.5 million workers.^4^

Workers in this sector — which heavily relieve on manual labor and often operate with poorly structured work processes — are exposed to various illnesses and accidents. These can harm their health and result in absenteeism, medical leave, or even death.^5^

A work accident is defined as any accident that happens while performing a job for a company or while insured individuals are working, resulting in a physical injury or functional impairment that leads to death, loss, or reduced ability to work.^6^ According to Law No. 8,213/1991, Article 22, all work-related or commuting accidents (those occurring while performing job duties or traveling between home and work) as well as occupational illnesses (caused or triggered by the job), must be reported by the employer to Social Security.^6^,^7^ The Work Accident Report (Comunicação de Acidente de Trabalho, CAT) is the official document used to report and recognize work-related accidents and occupational diseases. CAT is one of the main tools for collecting data and generating statistics on occupational health and safety.^8^

The construction industry is among the sectors with the highest risk of work-related accidents in Brazil. It ranks first for permanent disabilities, second for deaths, and fifth for absences longer than 15 days.^5^ NR-18 sets administrative, planning, and organizational guidelines aimed at implementing control measures and preventive safety systems in processes, working conditions, and the work environment in the construction industry.^9^

According to the latest Statistical Yearbook of Work Accidents (Anuário Estatístico de Acidentes do Trabalho, AEAT), in 2017, there were 549,405 work accidents across Brazil, of which 30,025 cases (5.46%) occurred in the construction industry. These cases were categorized as leave for less than 15 days, leave for more than 15 days, medical assistance, permanent disability, and deaths.^10^ The main challenges for implementing safety and health practices on construction sites are organizational culture, workers’ resistance to change, companies’ lack of knowledge, workers’ low education levels, and lack of commitment from leadership.^11^

When companies in the construction industry do not consider the impact of work accidents, they become vulnerable to the consequences of these accidents. In the short and medium term, this issue leads to lower productivity and future consequences for both the worker and the government, which has to cover the costs of accident-related benefits and medical treatment.^12^ Analyzing work accident data is therefore essential to ensure victims’ rights, support worker training, and help implement actions to prevent work accidents in the construction industry. This study is justified as an initial, exploratory step toward a more detailed analysis that could later outline the epidemiological profile of affected workers. This could help propose actions to improve the quality of life of construction workers in the future. The construction industry plays a key role in the economy of Santa Catarina, generating jobs, income, and contributing to the state’s social and economic development.^13^ Therefore, this study aims to answer the following guiding question: What was the temporal evolution of work accidents in the construction industry in Santa Catarina between 2011 and 2020?

## METHODS

This is an ecological observational study using time series analysis. The study population consisted of construction industry workers in the state of Santa Catarina who suffered work-related accidents between 2011 and 2020 and were covered by the General Social Security System (Regime Geral de Previdência Social, RGPS). Given the census nature of this study, no relevant cases were excluded, except for those classified as ignored or unavailable in the data tables.

Data were collected from the Social Security statistics—specifically, the Statistical Yearbooks of Social Security (Anuários Estatísticos da Previdência Social, AEPS) and the AEAT — which cover only formal workers in the economy. These represent about 30% of Brazil’s economically active population (EAP).

Data collection focuses on two dependent, qualitative, nominal, and multi-category variables. The first was the reason/situation (typical accident with CAT, commuting accident with CAT, occupational illness with CAT, and cases without CAT), related to accident and leave records. The second was consequences (medical assistance, disability for less than 15 days, disability for more than 15 days, permanent disability, and deaths), referring to benefits processed by Social Security during the study period. Additionally, data were collected on accidents in Classificação Nacional de Atividades Econômicas (CNAEs) categories related to the construction industry, including building construction – CNAE 41, infrastructure works – CNAE 42, and specialized construction services – CNAE 43. For the time trend analysis (time-event correlation), the independent variable was the study period (2011-2020), considered as a discrete qualitative variable.

This study was based on publicly available secondary data, with no identification of participants, using population aggregates as the unit of analysis. Therefore, it did not require approval by Human Research Ethics Committees, according to Resolution CNS No. 510/2016 (Article 1, items II, III, and V).

Data collection was carried out and organized using Infologo software and then exported to Excel® for rate calculations. Analyses were performed using SPSS 20.0 Statistical Product for Service Solutions (SPSS INC., Chicago, Illinois, USA). For rate calculations, the numerator was the number of accidents or consequences, and the denominator was the number of workers in the economic activity of interest. A constant of 1,000 was used, except for mortality rates, where a constant of 100,000 was applied.

For the temporal analysis of the rate series of the variables of interest, the following were calculated: the average rate of each series, Spearman’s correlation coefficient (time-event relationship), the average annual variation of the series values (β), calculated using Pearson’s linear regression, and the p-value obtained through analysis of variance (ANOVA). A p-value < 0.05 was considered statistically significant. The results were presented in absolute and proportional terms, and incidence was expressed as rates or coefficients.

## RESULTS

Table 1 shows that, during the study period, regarding work accidents in CNAE 41 classified by reason/situation, typical accidents with CAT were the most frequent, accounting for 5,296 cases (57.22%), with an average rate of 9.56 accidents per 1,000 workers. Accidents classified as “without CAT” ranked second, with 2,719 cases (29.38%) and an average rate of 4.91 accidents per 1,000 workers.

**Table 1:** Absolute frequency, relative frequency (%), and average rate (×1,000) of accidents that occurred and were processed in the construction industry (CNAEs 41, 42, and 43), according to the variables studied. Santa Catarina, 2011-2020

Variable	Frequency	%	Average rate (×1.000)
Reason/Situation (CNAE 41)	n = 9,256		
Typical – CAT	5,296	57.22	9.56
Commuting – CAT	1,186	12.81	2.14
Occupational disease – CAT	55	0.59	0.09
Without CAT	2,719	29.38	4.91
Consequences (CNAE 41)	n = 12,210		
Medical assistance	415	3.40	0.74
Disability < 15 days	5,522	45.23	9.97
Disability > 15 days	5,514	45.16	9.95
Permanent disability	674	5.52	1.21
Deaths	85	0.70	0.15
CNAE Division (41, 42, and 43)	n = 21,398		
Building construction	11,406	53.30	20.6
Infrastructure works	5,259	24.58	29.77
Specialized construction services	4,733	22.12	16.15

Source: Statistical Yearbook of Social Security (2011-2020), adapted by the authors.

CAT = Work Accident Report; CNAE = National Classification of Economic Activities.

Regarding the consequences of work accidents in building construction (CNAE 41), cases of disability lasting less than 15 days and those with more than 15 days of leave showed similar results, with 5,522 recorded cases (45.23%) and 5,514 cases (45.16%), and average rates of 9.97 and 9.95 accidents per 1,000 workers, respectively. When comparing occupational accidents across the CNAE divisions related to the construction industry, the building construction segment had the highest number of recorded accidents, with 11,406 cases (53.3% of the total). The infrastructure works segment registered 5,259 cases (24.58%), and specialized construction services reported 4,733 cases (22.12%). In terms of accident risk, the infrastructure works segment had a higher rate (29.77 accidents/1,000 workers) compared to building construction, which showed a rate of 20.6 accidents/1,000 workers.

Table 2 shows the incidence rates of work accidents recorded in Santa Catarina involving formal building construction workers, according to reason/situation, during the period analyzed. The rates of typical accidents showed a clear downward trend from 2011 to 2014, followed by a reversal of this trend between 2015 and 2020, when the rates began to increase. Commuting accidents showed relatively stable rates throughout the entire study period, as did occupational diseases (Spearman = 0.176 and -0.139, respectively).

**Table 2:** Work accident rates recorded under CNAE 41 (Building Construction) by year of occurrence and reason/situation, Santa Catarina, 2011-2020

Year\reason	With CAT			Without CAT	Total
	Typical WA	Commuting WA	Occupational disease		
2011	10.77	2.36	0.09	9.85	23.07
2012	9.71	2.12	0.10	9.77	21.70
2013	9.06	1.96	0.14	8.33	19.48
2014	9.46	2.10	0.14	0.11	11.81
2015	8.05	1.75	0.09	1.70	11.59
2016	8.53	2.26	0.07	3.53	14.39
2017	8.57	2.02	0.08	3.24	13.91
2018	9.27	2.10	0.02	4.25	15.65
2019	10.75	2.76	0.14	5.04	18.68
2020	12.61	2.21	0.12	2.12	17.06
Average	9.56	2.14	0.10	4.91	16.72
Spearman	0.079	0.176	-0.139	-0.455	-0.358
Beta	0.305	0.281	-0.081	-0.599	-0.394
p-value	0.391	0.431	0.825	0.067	0.259

Source: Statistical Yearbook of Social Security (2011-2020), adapted by the authors.

AT = work accident; CAT = Work Accident Report; CNAE = National Classification of Economic Activities.

The rates of accidents without CAT showed an irregular pattern. There was a downward trend during the first three years, followed by a sharp break in the historical series in 2014 (with a rate of 0.11 accidents per 1,000 workers). From these very low rates, there was an upward trend between 2015 and 2019, after which the rate dropped again. Considering all the data presented, it can be observed that all reasons (with CAT) and the category without CAT showed no clear temporal trend during the period analyzed (p > 0.05). Regarding work accidents processed in the building construction sector in Santa Catarina, by their consequences (outcomes), shown in Table 3, there was a significant upward trend in the more favorable outcomes — Medical Assistance and Leave due to disability for less than 15 days — with Spearman coefficients of 0.661 and 0.915 and p-values of 0.039 and < 0.001, respectively. Cases requiring medical assistance after the accidents showed a strong correlation, indicating an upward trend over the years analyzed.

**Table 3:** Rates of work accidents processed under CNAE 41 (Building Construction), by year of occurrence and consequence, Santa Catarina, 2011-2020

Ano	Medical assistance	Disability < 15 days	Disability > 15 days	Permanent disability	Deaths	Total
2011	0.359	7.866	18.468	1.265	0.222	28.180
2012	0.618	7.270	17.739	1.394	0.174	27.194
2013	0.881	7.400	15.697	1.641	0.167	25.786
2014	0.630	7.649	6.389	1.643	0.169	16.480
2015	0.567	9.612	4.814	0.153	0.169	15.315
2016	0.680	8.252	9.005	0.901	0.074	18.912
2017	0.857	10.628	7.507	1.244	0.122	20.358
2018	0.702	13.118	6.378	1.935	0.106	22.239
2019	0.752	16.313	6.653	1.663	0.137	25.518
2020	1.780	16.553	2.430	0.313	0.168	21.245
Average	0.750	9.973	9.959	1.217	0.154	22.052
Spearman	0.661	0.915	-0.745	-0.060	0.358	-0.976
Beta	0.657	0.899	-0.843	-0.176	-0.563	-0.315
p-value	0.039	0.000	0.002	0.628	0.090	0.376

Source: Statistical Yearbook of Social Security (2011–2020), adapted by the authors.

CNAE = National Classification of Economic Activities.

The less favorable outcomes — disability for more than 15 days and permanent disability — showed a significant downward trend (Spearman = -0.745; p = 0.002) and a tendency toward stability (Spearman = -0.060; p = 0.628), respectively. Regarding deaths, the occupational mortality rate showed no statistically significant temporal trend (Spearman = 0.358; p = 0.376).

Table 4 shows the rates of work accidents in the three CNAE divisions that make up the construction industry classification. Building construction was the only CNAE division that did not show a clear temporal trend during the study period (Spearman = -0.333; p = 0.397). The other divisions — infrastructure works (p = 0.029) and specialized construction services (p = 0.019) — showed well-defined temporal trends. The infrastructure works division showed a downward trend (Spearman = -0.612), while the specialized construction services division showed an upward trend in accident rates during the study period (Spearman = -0.527).

**Table 4:** Work accident rates recorded in the construction industry (CNAEs 41, 42, and 43), by year of occurrence and selected CNAEs, Santa Catarina, 2011-2020

Ano	Building construction CNAE 41	Infrastructure works CNAE 42	Specialized services CNAE 43	Total
2011	26.69	34.83	24.30	27.64
2012	25.55	34.28	20.87	25.94
2013	23.92	28.91	21.92	24.31
2014	14.70	29.84	13.29	16.74
2015	14.78	29.23	11.29	15.96
2016	17.88	25.74	14.62	18.17
2017	18.56	26.87	13.20	18.39
2018	20.16	27.45	14.50	19.74
2019	23.81	29.33	14.46	21.73
2020	20.72	28.66	13.89	19.77
Average	20.60	29.77	16.15	20.91
Spearman	-0.333	-0.612	-0.527	-0.345
Beta	-0.302	-0.686	-0.718	-0.537
p-value	0.397	0.029	0.019	0.109

Source: Statistical Yearbook of Social Security (2011-2020), adapted by the authors.

CNAE = National Classification of Economic Activities.

## DISCUSSION

This study used quantitative data from the AEPS and the AEAT, published by Social Security. These data allowed for a temporal analysis of work accidents in the building construction sector in the state of Santa Catarina, based on the CNAE. The analysis focused on the reason/situation of the accidents, the CNAE divisions where the accidents occurred, and the consequences of the processed accidents.

The economic crises that affected Brazil in recent years had a direct impact on the evolution and performance of the construction industry. This sector acts as one of the key indicators of the economy, quickly reflecting the effects of periods of recession, stagnation, or economic growth.^14^ Data (Table 2) showing the evolution of work accident risk by reason/situation in building construction in the state of Santa Catarina from 2011 to 2020 indicate that none of the variables studied during this period showed a clear trend (Table 2). The recurring economic crises directly impacted hiring practices and, as a result, affected accident rates by reason/situation. The analyzed rate series demonstrate that the risk of accidents increased in certain years and decreased in others, reflecting the volatility of the market in response to Brazil’s economic situation during the study period.^14^

**Figure 1:**
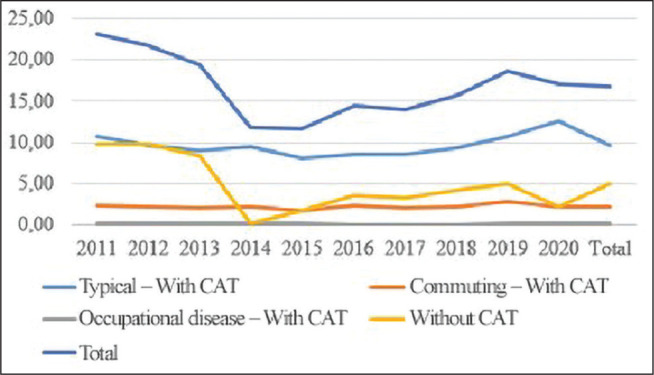
Graph of work accident rates recorded in building construction, by reason/situation and year of occurrence, Santa Catarina, 2011-2020. CAT = Work Accident Report.

Regarding accidents classified as “Without CAT,” there was a significant reduction during the period analyzed (Figure 1). This classification is directly related to the application of a mechanism called Social Security Epidemiological Technical Nexus (Nexo Técnico Epidemiológico Previdenciário, NTEP). This is a tool used by Social Security to systematically evaluate and filter leave requests submitted to the National Institute of Social Security (Instituto Nacional do Seguro Social, INSS) by companies, verifying whether they meet the criteria for collective risk (epidemiological nexus) or not.

Through the NTEP filter, many leave cases that were previously classified as unrelated to work due to the absence of a CAT (coded as B31 – social security nexus) were reclassified as “Without CAT” but now recognized as work-related (B91). Established by Law no. 11,430 of December 26, 2006,^15^ this allowed INSS medical evaluations to determine the work-related nature of disabilities based on the occurrence of NTEP, by assessing the connection between the company’s activity (CNAE) and the condition causing disability, as defined in the International Classification of Diseases (ICD).

The NTEP was added to the list of accident-related classifications, removing the need for a CAT to establish the work-related nature of the case.^16^ In 2007, the National Confederation of Industry (Confederação Nacional da Indústria, CNI) filed a Direct Action of Unconstitutionality (ADI No. 3,931/2007) against the implementation of the NTEP. After nearly 13 years of proceedings in the Brazilian Federal Supreme Court (*Supremo Tribunal Federal*, STF), the action was dismissed in 2020, confirming the constitutionality of the NTEP. This method has since made it possible to identify connections between work and health that were previously not recognized, encouraging broader knowledge of these relationships through their mapping and quantification.^16^-^21^ It has also helped generate new hypotheses for further etiological investigation. However, the way the NTEP establishes the epidemiological nexus for social security purposes requires additional studies to validate its effectiveness and to ensure it captures the dynamic reality of work, aiming for its continuous improvement and updating.

Regarding the consequences of work accidents in building construction, there was a trend of increasing rates of short-term disability leave (less than 15 days). One possible explanation for this rise in less severe accident rates is the creation of economic mechanisms that encourage companies to adopt measures to improve workplace safety, such as the Social Security Accident Factor (Fator Acidentário Previdenciário, FAP), which has been in effect since 2010. Since then, companies have been either penalized or rewarded depending on whether their work accident rates are above or below the average for their economic sector. The frequency, severity, and cost of these accidents directly affect the calculation of the company’s FAP, as established by Article 10 of Law No. 10,666/2003.^22^

The FAP adjusted the rates for Work Accident Insurance (Seguro de Acidente do Trabalho, SAT), which are set at 1%, 2%, or 3% of the company’s payroll, depending on the company’s risk level. These rates are multiplied based on the severity of the damage and the economic impact of work accidents that occurred in each company, to encourage companies to improve working conditions.^23^,^24^ Companies with the lowest levels of morbidity and severity can receive up to a 50% reduction in SAT payments.^23^,^24^

On the other hand, if a company shows a higher rate of work accidents and its social security morbidity indicators place it among the worst-performing companies in its economic sector, the rates applied to its payroll for Workplace Environmental Risks (Riscos Ambientais de Trabalho, RAT) — the new name for SAT — can be doubled.^24^,^25^ The analysis of processed accidents also showed a downward trend in the outcomes “Medical Assistance” and “Disability for more than 15 days” (Table 3). Thus, the FAP once again appears as a plausible explanation for the apparent reduction in workplace risks, encouraging companies to adopt more effective occupational safety measures.

With the reduction in accidents causing longer temporary disability and the increase in those associated with shorter disability periods, there was a reversal in the rates of leave for more than 15 days compared to those with less than 15 days.

The NRs undoubtedly play a key role in promoting workplace safety, especially NR-18 and NR-35. NR-18 is the main tool for preventing accidents in the construction industry. It aims to establish administrative, planning, and organizational guidelines for implementing control measures and preventive safety systems in work processes, conditions, and environments. NR-35 focuses on managing safety and health in work at heights. It sets requirements to protect workers from risks associated with tasks performed at different levels, particularly regarding fall prevention. Depending on the complexity and risks of these activities, employers must adopt additional safety measures.^26^

Together with the Conduct Adjustment Terms (Termos de Ajustamento de Conduta, TACs), issued when companies are fined by the Brazilian Labor Public Prosecution Office (Ministério Público do Trabalho, MTP), for problems in the work environment, these regulations can help increase companies’ accountability in ensuring the safety and well-being of workers. These responsibilities are reinforced by the commitments made with the MPT to comply with the rules related to workplace hygiene, health, and safety.

The efforts of construction companies to use safer, more efficient equipment to perform construction work should be highlighted, such as the replacement of hoist elevators with rack-and-pinion elevators by most construction companies.^27^

When comparing work accident rates across the CNAE divisions commonly known as the “construction industry” between 2011 and 2020, the CNAE 41 division (building construction) recorded the highest number of work accidents during the analyzed period (53.3% of the total, with an average rate of 20.60 accidents/1,000 workers). However, when looking at the average incidence rates by CNAE division, the division with the highest accident rate was infrastructure works (CNAE 42), with 29.77 accidents/1,000 workers, indicating a 1.44 times higher relative risk compared to the building construction division.

Infrastructure works, which mostly involve large-scale projects, tend to pose greater risks to the workers involved. This is due to the higher frequency of working at heights and the more challenging working conditions, often outdoors and, in many cases, without the possibility of setting up fully safe construction sites. Additionally, these projects require qualified supervision teams to oversee all stages of execution, which, at times, is not adequately provided.^28^

Workers involved in road infrastructure projects are exposed to injury risks caused by the movement of construction vehicles and equipment within work zones, in addition to the traffic of vehicles often passing at high speeds. These workers, regardless of their specific activities, are also exposed to poor lighting conditions, limited visibility, and various weather conditions.^28^ This branch of the construction industry shows a continued upward trend in the number of accidents, reflecting both a certain resilience during periods of economic crisis and ongoing difficulties in implementing effective safety measures.

In contrast, there was a considerable reduction in the number of work accidents in building construction in Santa Catarina during the analyzed period. This positive scenario can be partly attributed to the set of incentive measures implemented by the Brazilian Federal Government, and partly to the impact of the economic instability during the study period, which forced the sector to reduce its workforce by almost 50% compared to the pre-crisis period in 2014.

## CONCLUSIONS

This study allowed for an analysis of the temporal evolution of work accidents in the construction industry in Santa Catarina between 2011 and 2020. The results show that, for building construction, the occurrence rates of work accidents for most of the reasons analyzed remained relatively stable over time, except for accidents classified as “Without CAT,” which showed an irregular pattern but with a significant reduction during the study period.

Regarding processed accidents, the analysis of the consequences of work accidents in building construction revealed an upward trend in the rate of leave due to disability lasting less than 15 days, and a reduction in the rates of disability with leave of 15 days or more, indicating a relative decrease in the severity of accidents within the CNAE division analyzed.

Finally, the comparison of accident rate trends among the three different CNAE divisions that make up what is commonly referred to as the construction industry showed that CNAE 41 (building construction) was the division with the highest number of work accidents during the analyzed period, accounting for 53.3% of the total records. However, the division with the highest average accident rate was CNAE 42 (infrastructure works), indicating a 1.44 times higher relative risk compared to the building construction division.

As a background scenario, the analyzed period was heavily influenced by an initial phase of economic growth (2011-2014), followed by a significant recession period (2015-2019), and ended with the recession effects caused by the COVID-19 pandemic in 2020 — all of which had a direct impact on the health and safety of workers in this dynamic (and sensitive) sector of the Brazilian industry.
